# Potential Immunomodulatory Properties of Biologically Active Components of Spices Against SARS-CoV-2 and Pan β-Coronaviruses

**DOI:** 10.3389/fcimb.2021.729622

**Published:** 2021-08-27

**Authors:** Sourodip Sengupta, Debina Bhattacharyya, Grishma Kasle, Souvik Karmakar, Omkar Sahu, Anirban Ganguly, Sankar Addya, Jayasri Das Sarma

**Affiliations:** ^1^Department of Biological Sciences, Indian Institute of Science Education and Research Kolkata (IISER-K), Mohanpur, India; ^2^Kimmel Cancer Centre, Thomas Jefferson University, Philadelphia, PA, United States

**Keywords:** COVID-19, hyperinflammation, hypercytokinemia, spices, bioactive components, antioxidants, Nrf2

## Abstract

The severe acute respiratory syndrome coronavirus 2 (SARS-CoV-2)-induced COVID-19 has emerged as a defining global health crisis in current times. Data from the World Health Organization shows demographic variations in COVID-19 severity and lethality. Diet may play a significant role in providing beneficial host cell factors contributing to immunity against deadly SARS-CoV-2 pathogenesis. Spices are essential components of the diet that possess anti-inflammatory, antioxidant, and antiviral properties. Hyperinflammation, an aberrant systemic inflammation associated with pneumonia, acute respiratory failure, and multiorgan dysfunction, is a major clinical outcome in COVID-19. Knowing the beneficial properties of spices, we hypothesize that spice-derived bioactive components can modulate host immune responses to provide protective immunity in COVID-19. This study emphasizes that biologically active components of spices might alleviate the sustained pro-inflammatory condition by inhibiting the activity of tumor necrosis factor-alpha (TNF-α), interleukins (IL6, IL8), and chemokine (CCL2) known to be elevated in COVID-19. Spices may potentially prevent the tissue damage induced by oxidative stress and pro-inflammatory mediators during SARS-CoV-2 infection. The current study also highlights the effects of spices on the antioxidant pathways mediated by Nrf2 (nuclear factor erythroid 2-related factor 2) and Hmox1 (heme oxygenase 1) to restore oxidative homeostasis and protect from aberrant tissue damage. Taken together, the anti-inflammatory and antioxidant activities of bioactive components of spices may hold a promise to target the cellular pathways for developing antivirals against SARS-CoV-2 and pan β-coronaviruses.

## Introduction

The COVID-19 pandemic caused by the severe acute respiratory syndrome coronavirus 2 (SARS-CoV-2) (Zhu et al., 2020) is the third case of zoonotic transmission of coronaviruses (CoVs) in the human race after SARS-CoV (in 2003) (Peiris et al., 2003) and Middle East respiratory syndrome coronavirus (MERS-CoV; in 2012) (Zaki et al., 2012). According to WHO COVID-19 Dashboard (01 June 2021), the global confirmed cumulative cases had crossed 170 million, and more than 3.6 million cumulative deaths were reported with significant varied demographic distributions (World Health Organization, 2021a). Multidimensional factors such as population density, age, obesity, comorbidity, seasonality, temperature, humidity, social distancing, and critical care capacity have played a significant role in deciding COVID-19 disease incidence and mortality (Kissler et al., 2020; Cao et al., 2021). Among several other possible factors that may be significant is diet (Bousquet et al., 2020). Dietary fats and fibers can influence gut microbiota composition, altering the immune response and susceptibility to respiratory distress as prevalent in SARS-CoV-2 (Wypych et al., 2017).

In this context, it is valuable to integrate knowledge from previous experiences with infectious viruses like influenza, West Nile, dengue, HIV, and human coronaviruses (HCoVs) to repurpose drugs. While other viruses and their pathogenesis are well studied, little information is available about human beta-coronaviruses. So far, seven human CoVs (HCoVs) have been identified. Among the HCoVs, the α-CoVs (NL63 and 229E) and the β-CoVs (OC43 and HKU1) infect the upper respiratory tract and only cause mild common cold symptoms (Weiss, 2020). The β-CoVs SARS-CoV, MERS-CoV, and SARS-CoV-2 can infect the lower respiratory tract and cause a severe acute respiratory syndrome with high zoonotic potentials making their study difficult (Peiris et al., 2003; Zaki et al., 2012; Wu et al., 2020; Zhu et al., 2020). The periodic emergence of the new CoVs among humans can be attributed to their great genetic diversity, recurrent genetic recombination, and cross-species transmission due to increased human-animal interaction (Peiris et al., 2003; Zaki et al., 2012; Weiss, 2020; Wu et al., 2020; Zhu et al., 2020).

COVID-19 is considered a respiratory disease as SARS-CoV-2 primarily targets the respiratory system. Clinical reports show that direct lung damage in COVID-19 patients is related to the development of acute pneumonia, diffused alveolar pathology associated with massive infiltration of neutrophils and macrophages, and edema in alveolar walls (Martines et al., 2020; Xu et al., 2020). Interestingly, SARS-CoV-2 also infects other major organs such as the nervous system, cardiovascular system, liver, gastrointestinal tract, and kidneys (Gu et al., 2005; Mao et al., 2020; Mokhtari et al., 2020).

**Figure 1:**
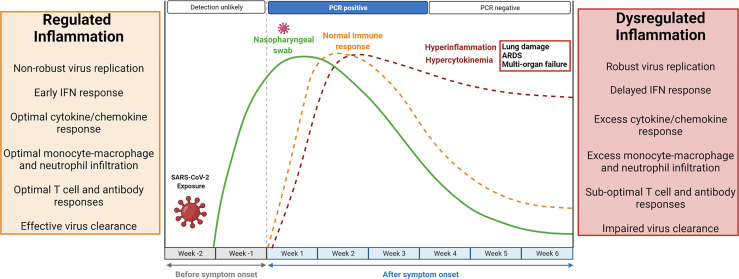
Kinetics of SARS-CoV-2 replication and the antiviral immune response. The median incubation period of SARS-CoV-2 is 4-5 days, and symptoms usually start appearing by 11.5 days. Within a week of symptoms appearing, viral load reaches its peak and becomes detectable in RT-PCR tests. A non-robust viral replication is associated with a regulated and protective immune response. Early IFN (interferon) response, optimal monocyte-macrophage and neutrophil infiltration, optimal secretion of pro-inflammatory cytokines and chemokines, and optimal lymphocyte responses result in effective viral clearance. The inflammatory response resolves within 4-5 weeks after symptom appearance, viral particles go below the detection limit, but viral RNA may persist in low amount, and the person recovers. However, robust SARS-CoV-2 replication may lead to delayed IFN response, excess monocyte-macrophage and neutrophil infiltrations, sub-optimal lymphocyte response, and impaired viral clearance. The resulting aberrant inflammatory response known as hyperinflammation is associated with excessive secretion of pro-inflammatory cytokines and chemokines (hypercytokinemia) and accumulation of inflammatory cells in the lungs. Hyperinflammation and hypercytokinemia may lead to diffused alveolar damage (DAD), acute respiratory distress syndrome (ARDS), and multiorgan failure. Created with BioRender.com.

Inflammation is a vital part of the host response to infection. Successful elimination of any infection requires a well-coordinated inflammatory response consisting of several elements comprising T lymphocytes efficient in killing infected cells, macrophages able to phagocytose foreign antigen (viruses), and antibodies that neutralize viruses (Medzhitov, 2008). A non-robust replication of SARS-CoV-2 in the airway epithelial cells initiates an early IFN (interferon) response, optimal infiltration of monocyte-macrophages, neutrophils, and lymphocytes associated with optimal secretion of pro-inflammatory cytokines and chemokines. This results in effective elimination of infected cells, blocking of viral infection, and timely recovery (Channappanavar and Perlman, 2017; Tay et al., 2020). In contrast, a robust replication of SARS-CoV-2 leads to delayed IFN response and profuse infiltration of monocyte-macrophages and neutrophils, resulting in an uncontrolled local and systemic inflammatory response known as hyperinflammation (Channappanavar and Perlman, 2017; Coperchini et al., 2020; Tay et al., 2020). The kinetics of SARS-CoV-2 infection and the immune response is depicted in **Figure 1**. Data indicates that SARS-CoV-2 infection shares certain immunological features of SARS-CoV and MERS-CoV infection in terms of the cytokine and chemokine response (Coperchini et al., 2020). Clinical studies have shown elevated inflammatory cytokines, including tumor necrosis factor-alpha (TNF- α), interleukins (IL; IL2, IL6, IL7, IL8, IL9, and IL10), chemokines (CXCL10, CCL2, IP10, MCP1), and colony-stimulating factors (G-CSF, GM-CSF) in COVID-19 patients compared to a healthy person. Levels of these pro-inflammatory cytokines get further increased in hospitalized critical COVID-19 patients (Coperchini et al., 2020; Li et al., 2020). This phenomenon of high levels of inflammatory cytokines and chemokines, termed hypercytokinemia, is associated with increased disease severity and poor prognosis (Coperchini et al., 2020). Furthermore, hypercytokinemia is linked to life-threatening complications, including acute respiratory distress syndrome (ARDS), septic shock, and multiple organ dysfunction, which are the primary cause of death in COVID-19 patients. ARDS has an estimated 40% mortality rate, characterized by inflammatory injury to the alveolar-capillary membrane, bilateral lung infiltrations, and severe hypoxemia, leading to respiratory failures (Coperchini et al., 2020; Batah and Fabro, 2021). The SARS-CoV-2 infection affects the hematopoietic system causing lymphocytopenia and leukopenia (Gu et al., 2005; Mokhtari et al., 2020). The depletion of T lymphocytes (CD4+ and CD8+) and natural killer cells can increase the risk of bacterial infection. Lymphocytopenia can be attributed to the presence of ACE2 (angiotensin-converting enzyme 2) receptor on the lymphocytes and direct infection of these cells. Also, elevated levels of cytokines and chemokines may induce cellular apoptosis (Mokhtari et al., 2020).

The pathophysiological and clinical features of COVID-19 share many similarities with sepsis. Sepsis is a life-threatening disease syndrome caused by a systemic and dysregulated inflammatory response to the invasion of the bloodstream by pathogens (viruses, fungi, bacteria, or parasites). It is a significant cause of maternal and neonatal deaths (World Health Organization, 2021b). A recent study estimated 48.9 million cases and 11 million deaths were associated with sepsis in 2017, accounting for nearly 20% of all global deaths. Around 85% of sepsis-related cases and deaths worldwide are reported in low- and middle-income countries (Rudd et al., 2020). Although significant geographical differences exist in COVID-19 cases and mortality, the trend is opposite to what was observed in sepsis. South East Asia reported the third-highest number of COVID-19 cases until 01 June 2021, but deaths due to COVID-19 were relatively low compared to neighboring regions. We used the WHO COVID-19 data (World Health Organization, 2021a) to assess the fatality rate (the number of deaths divided by the number of confirmed cases multiplied by 100) as of 01 June 2021. The fatality rate in South East Asia was the lowest at 1.27% compared with fatality rates of 2.48% and 2.45% in Africa and Americas, respectively, 2.11% in Europe, 1.99% in Eastern Mediterranean, and 1.5% in the Western Pacific region. However, there are pitfalls associated with counting COVID-19 cases and deaths. Differences can be attributed to several factors such as less testing, whether deaths outside hospital counted, healthcare infrastructure, and reporting methods adopted by countries. When sepsis happens, it is challenging to diagnose, and many patients were categorized as critical septic patients and may not be counted as COVID-19 positive cases.

Sepsis is characterized by a dysregulated immune response, causing increased pro-inflammatory mediators (hypercytokinemia), leading to pathological inflammatory disorders. Clinical manifestations include vascular microthrombosis, hemolytic anemia, consumptive thrombocytopenia, disseminated intravascular coagulation, leukopenia, leukocytosis, cardiovascular and respiratory failure, multiorgan dysfunction, high predisposition to secondary infections, and septic shock. Strikingly, these clinical features are also common to COVID-19 (Li et al., 2020; Olwal et al., 2021). The complex clinical symptoms and pathological consequences make COVID-19 treatment a big challenge. Worldwide trials are ongoing to combat COVID-19 by taking insights from other infectious and inflammatory diseases. Simultaneously, efforts are put forward to understand the viral genome to design therapeutics and boost host immunity. In this regard, available and ongoing therapeutic options in the management of COVID-19 include antiviral drugs, anti-SARS-CoV-2 monoclonal antibodies, anti-inflammatory, and immunomodulatory agents (Cascella et al., 2021; Sethi and Bach, 2020). Currently, remdesivir, the broad-spectrum antiviral drug that has previously exhibited inhibitory effects on SARS-CoV-2 *in vitro* (Wang et al., 2020), is the only FDA-approved antiviral drug for COVID-19 treatment. Convalescent plasma therapy is a promising anti-SARS-CoV-2 neutralizing antibody therapy that has received EUA (emergency use authorization) from FDA for patients with life-threatening COVID-19 (U.S. Food & Drug Administration, 2021a). Potent anti-spike neutralizing monoclonal antibodies such as REGN-COV2 (a cocktail of two IgG1 antibodies), bamlanivimab, etesevimab, and sotrovimab have been approved by FDA under EUA (Gottlieb et al., 2021; U.S Food & Drug Administration, 2021b; Weinreich et al., 2021). Immunomodulatory agents such as corticosteroids (dexamethasone) have demonstrated promising results in a randomized trial by significantly lowering mortality in COVID-19 patients in critical conditions needing oxygen or ventilation support (Group et al., 2021). Dexamethasone is currently being administered either alone or combined with remdesivir in hospitalized COVID-19 patients requiring respiratory support. Anti-IL6 receptor monoclonal antibodies (tocilizumab) or IL6 receptor antagonists (Sarilumab and Siltuximab) are other immunomodulators currently in clinical trials for efficacy and safety checks (Investigators et al., 2021). Clinical trials are ongoing to check the effectiveness of recombinant human ACE2 (rhACE2) in inducing direct (via RAAS homeostasis) or indirect (chimeric receptor effect) beneficial results in COVID-19 (National Institutes of Health, 2021). Baricitinib, a selective inhibitor of Janus kinase (JAK 1 and JAK 2), has been approved for use in combination with remdesivir in hospitalized COVID-19 patients under a EUA issued by the FDA (Kalil et al., 2021). Clinical trials involving other JAK inhibitors (Ruxolitinib and Tofacitinib) or tyrosine kinase inhibitors (acalabrutinib, ibrutinib, rilzabrutinib) that regulate cytokine signaling and macrophage activation are currently being evaluated for use in COVID-19 treatment. Additional immunomodulators proposed are interferon-β-1a (IFN- β-1a) and interleukin (IL)-1 antagonists (Cascella et al., 2021). Given the lack of sufficient data regarding the efficacy of these agents, these are not currently recommended to treat COVID-19 infection.

Significant progress made in clinical research globally has led to a better understanding of the SARS-CoV-2 pathogenesis and resulted in the fast development of novel vaccines. Two mRNA-based vaccines, BNT162b2 (developed by BioNTech/Pfizer) and mRNA-1273 vaccine (Moderna) (Polack et al., 2020; Baden et al., 2021), have already received EUA approval from FDA and are currently being administered in several countries. A third vaccine, Ad26.COV2.S, based on adenovirus vector encoding full-length SARS-CoV-2 spike protein, has also received EUA by FDA (Sadoff et al., 2021). The ChAdOx1 nCoV-19 vaccine (Oxford/AstraZeneca) has been approved for emergency use in many countries but has not yet received FDA approval (Voysey et al., 2021). In addition, indigenously developed vaccines such as Sputnik V (Russia), Covaxin (India), and CoronaVac (China) have been granted emergency use in many countries. Another vaccine, NVX-CoV2373(Novavax), a recombinant SARS-CoV-2 nanoparticle genetically engineered vaccine, has shown promising results in clinical trials (Shinde et al., 2021). The emergence of SARS-CoV-2 variants, rising concerns regarding the efficacy of these vaccines against the new variants, the high zoonotic potential of HCoVs, and its repeated occurrences warrant pan-CoV therapy considering the common properties of CoVs and or should be driven to boost our immune system through dietary factors, which is the major focus of the current study.

Natural immunosuppressants in dietary sources (Peter et al., 2020; Alhazmi et al., 2021) and bioactive components in spices (Gupta et al., 2020; Devan et al., 2021; Kunnumakkara et al., 2021; Rajan et al., 2021) might offer a novel therapeutic and practical approach to fighting against COVID-19 and pan-CoV infection by providing protective immunity. Dietary components, as discussed, can provide antiviral effects by reducing oxidative stress and balancing pro-inflammatory and or anti-inflammatory mediators to maintain cellular and immune homeostasis. Thus, protecting the cells from oxidative damage and aberrant tissue damage, as evident in SARS-CoV-2 patients (Bousquet et al., 2020; Gupta et al., 2020; Peter et al., 2020; Alhazmi et al., 2021; Devan et al., 2021; Kunnumakkara et al., 2021; Rajan et al., 2021).

## Bioactive Components of Spices Against SARS-CoV-2-Induced Hyperinflammation

It has been suggested that fermented vegetables and milk products could be linked to the low prevalence of COVID-19 deaths in some European countries, Korea and Taiwan (Bousquet et al., 2021). Ecological studies have shown that an increase in each g/day consumption of fermented foods or cruciferous vegetables (cabbages) decreased COVID-19 mortality by a factor of 35.4% or 11-13.6%, respectively (Fonseca et al., 2020; Fonseca et al., 2020). Many fermented foods contain live microorganisms that can influence our gut microbiota (De Filippis et al., 2020), and a possible role of gut microbiota in COVID-19 has been implicated (Dhar and Mohanty, 2020). ACE2 serves as a SARS-CoV-2 receptor, and the binding of SARS-CoV-2 to ACE2 results in oxidative stress (Ji et al., 2020). Components in fermented cabbages and milk products have potent antioxidant and anti-ACE activity (Ahtesh et al., 2018; Dang et al., 2019; Gharehbeglou and Jafari, 2019). A recent study examined the daily spice consumption per capita across 163 countries affected with COVID-19 and observed a correlation between COVID-19 cases per million population tested and spice consumption per capita per day (Elsayed and Khan, 2020). Countries with lower spice consumption showed more COVID-19 cases and deaths per million population than those with higher daily consumption. Spices have been used as preservatives, flavoring agents in food, and as medicines for centuries. Over the years, research into their beneficial health effects has gained momentum as spices aid in preventing and treating various chronic diseases (Bi et al., 2017; Kunnumakkara et al., 2018). To date, several works have been published associating spices with disease severity and mortality in COVID-19 (Gupta et al., 2020; Bousquet et al., 2021; Devan et al., 2021; Kunnumakkara et al., 2021; Rajan et al., 2021; Singh et al., 2021; Vicidomini et al., 2021), but the possible mechanisms of immune-modulatory action of spices remain elusive.

To better elucidate the cytoprotective effects of spices, it is important to understand which food components can be considered spices. Spices are aromatic parts of plants used in whole, broken, or ground form mainly for seasoning. Different dried parts of plants rich in volatile oils and aromatic scents are used as spices such as roots (turmeric); barks (cinnamon); seeds (cumin); buds or flowers (clove, saffron); leaves (bay leaves); and fruits or berries (chili, black pepper). Spices have been shown to possess antimicrobial, anti-inflammatory, antioxidant, and antiviral properties (Rubio et al., 2013; Liu et al., 2017; Kunnumakkara et al., 2018; Yan et al., 2019). Bioactive components in spices such as curcumin (the active ingredient of turmeric) and cinnamaldehyde (cinnamon) block the replication of influenza viruses *in vitro* and *in vivo* (Hayashi et al., 2007; Han et al., 2018). In addition, curcumin regulates the immune response by inhibiting cytokine production in macrophages upon influenza virus infection (Han et al., 2018). Luteolin (asafoetida) and quercetin (fennel seeds) have previously demonstrated anti-SARS-CoV activities by interfering with the virus entry into the host cells (Yi et al., 2004). Fresh ginger extracts have shown antiviral activity against the human respiratory syncytial virus by blocking virus attachment and internalization in mucosal cell lines (Chang et al., 2013).

**Figure 2:**
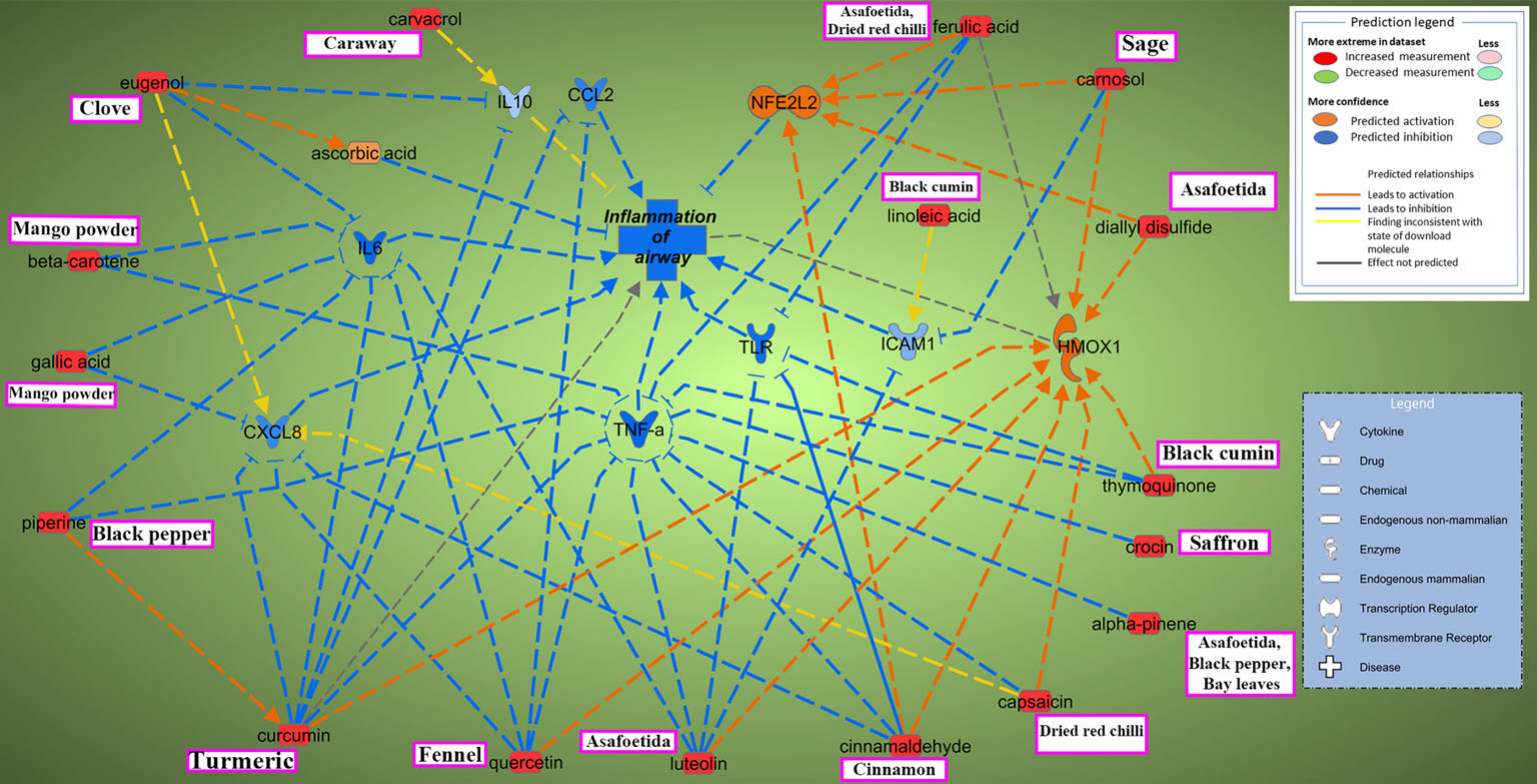
Ingenuity Pathway Analysis reveals interaction between bioactive components in spices and inflammation of the airway. Two possible mechanisms depicted through which active components from commonly used spices could help mitigate airway inflammation in COVID-19. One pathway involves inhibiting major pro-inflammatory cytokines and chemokines such as tumor necrosis factor-alpha (TNF-α), interleukins (CXCL8 (IL8), IL6, IL10), and chemokine CCL2; blocking inflammatory signal onset by inhibiting TLR (toll-like receptors) or inhibiting ICAM-1 (intercellular adhesion molecule 1) dependent migration of immune cells to the site of inflammation. Another way bioactive components could maintain inflammatory homeostasis is by targeting the oxidative stress pathway *via* activation of NFE2L2 (nuclear factor erythroid 2-related factor 2; Nrf2) and HMOX1(heme oxygenase 1). Synergistic effects of spices could also be evaluated, for example, between piperine and curcumin in attenuation of COVID-19 linked hyperinflammatory response. The name of major spice source(s) for the bioactive components are mentioned in boxes (pink). Interactive network constructed using Ingenuity Pathway Analysis (IPA) software (Qiagen Inc., Germantown, MD). Arrow-head indicates activation; bar-head indicates inhibition. Prediction legend indicates the relationship between molecules.

**Figure 3:**
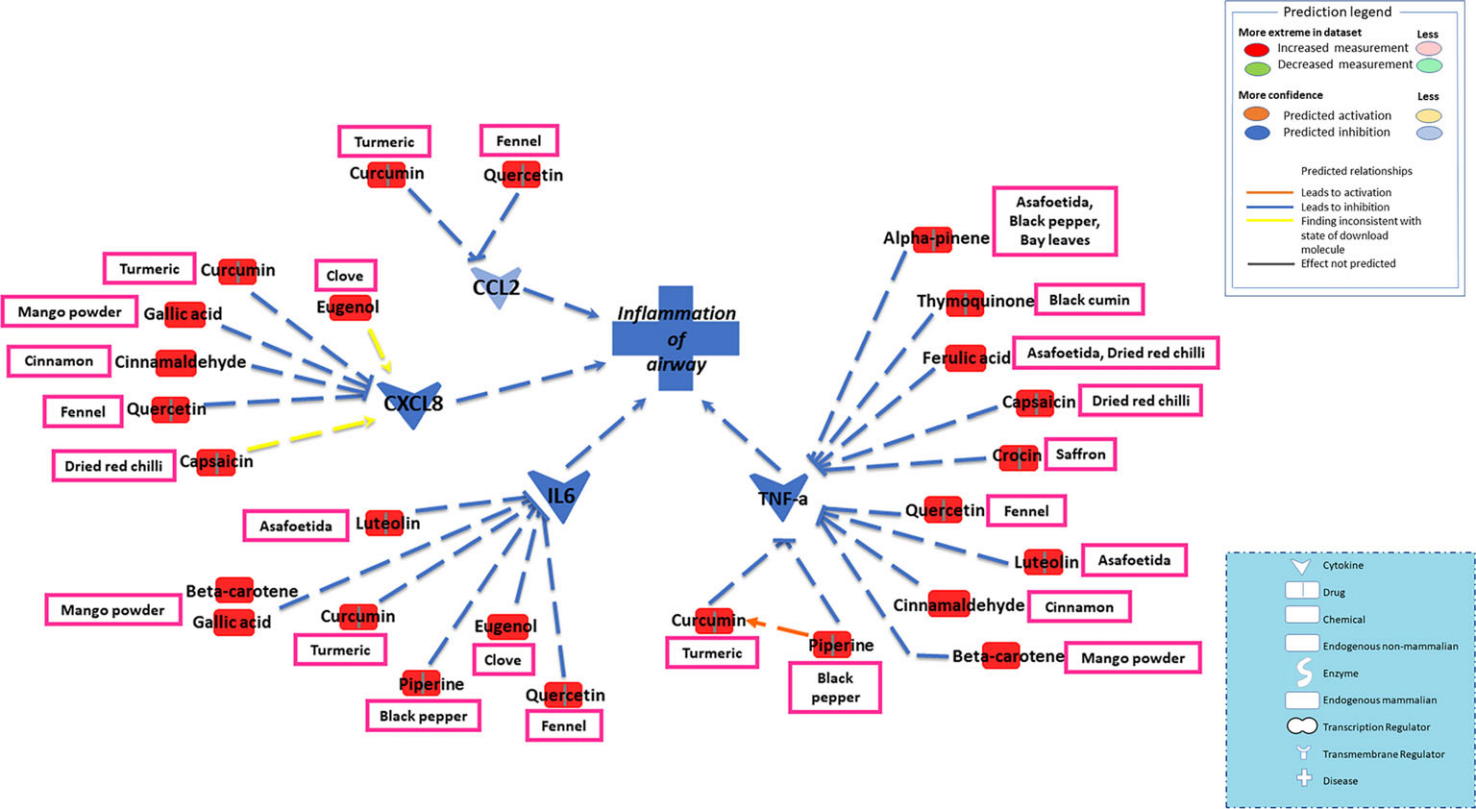
Network depicting direct or indirect interactions between bioactive components in spices and key inflammatory mediators such as TNF- α, CXCL8 (IL8), IL6, and CCL2 known to be elevated in COVID-19 patients. Hypercytokinemia in COVID-19 patients is associated with elevated levels of inflammatory cytokines, including tumor necrosis factor-alpha (TNF- α), interleukins (IL6, IL8), and chemokine CCL2. Levels of these pro-inflammatory cytokines get further increased in hospitalized COVID-19 patients. Based on the IPA data, a focused view on the interaction among these cytokines and bioactive components is depicted. The bioactive components can predictably inhibit TNF- α, CCL2, CXCL8 (IL8), and IL6. Further, the IPA network demonstrates the synergistic effects between most of the bioactive components and multiple inflammatory mediators. Piperine and curcumin cross-talk that enhance their downstream effects in TNF-α inhibition depicted in the IPA network.

The current study focused on the role of spices in the management of COVID-19. While the type of diet followed varies geographically, spices are used more or less in all culinary cultures. This study explores the mechanism of action of spices in COVID-19 by targeting possible airway inflammatory pathways. Spices common in the South-Asian region and known globally were selected based on two criteria: 1) availability of information about its bioactive components; 2) knowledge about antimicrobial, antiviral, or anti-inflammatory properties of bioactive components *in vitro* and *in vivo* studies. In this study, Ingenuity Pathway Analysis software (IPA; Qiagen Inc., Germantown, MD) was used to construct networks showing the potential direct or indirect interactions between bioactive components in spices (**Table 1**) and different cellular factors and cellular sensors, leading to downstream oxidative stress and inflammatory pathways. Only those compounds for which IPA predicted involvement in the airway inflammation were chosen finally. The list of selected spices and bioactive components is provided in **Table 1**. Network analysis using IPA suggests that most of the bioactive components of spices (**Table 1**) such as gallic acid, beta-carotene, eugenol, ferulic acid, curcumin, cinnamaldehyde, alpha-pinene, and diallyl disulfide can interact with the mediators of inflammation such as TNF- α, CXCL8 (IL8), IL6, IL10, TLR, ICAM-1, and CCL2 and inhibit their function as shown in **Figure 2**. After the entry of the virus into the host cell, pathogen recognition receptors like TLRs (toll-like receptors) present on epithelial cells and alveolar macrophages identify the virus and trigger pro-inflammatory signal transduction (Medzhitov, 2008; Garcia, 2020). Recognition of SARS-CoV-2 virus particles by TLRs results in the release of IL-1β, IL6, and TNF-α that induces lung inflammation and fibrosis (Zheng et al., 2021). Cytokines and chemokines attract innate immune cells, including natural killer (NK) cells, dendritic cells, monocytes, and polymorphonuclear leukocytes, which recruit lymphocytes. The adaptive immune response mediated by CD4+ T-cells signals antibody-secreting B-cells and cytotoxic CD8+ T-cells capable of recognizing and eliminating the virus. SARS-CoV-2 infection activates both innate and adaptive immune responses (Tay et al., 2020). TNF- α is a central pro-inflammatory cytokine in viral diseases, and blocking TNF- α or its receptor decreases SARS-CoV-associated disease severity and mortality in mice (McDermott et al., 2016; Coperchini et al., 2020). The CXCL8 (IL8) levels are elevated in the plasma of ARDS patients and serve as a potential prognostic biomarker for disease outcomes. Due to its chemoattractant activity towards neutrophils and monocytes, CXCL8 also plays a central role in the inflammatory response to respiratory tract infection (Coperchini et al., 2020). A study conducted in 43 adult COVID-19 patients showed high levels of IL6 could be positively related to disease severity (Gao et al., 2020). As depicted in **Figure 3**, cytokines such as TNF- α, CCL2, CXCL8, and IL6, which show high levels in COVID-19 patients requiring ICU admission (Li et al., 2020), can predictably be inhibited by the bioactive components. Also, the IPA network suggests that most bioactive components may act on multiple inflammatory mediators synergistically that need experimental validation. Furthermore, the bioactive components could also downregulate transcription of pro-inflammatory genes by regulating the NFκB pathways at multiple stages; suppressing IKKβ activation, inhibiting the degradation of IκBα, downregulating p65, and blocking the translocation of NFκB p65 into the nucleus (Lang et al., 2004; Lee et al., 2015; Cheng et al., 2019).

**Table 1:** Common and scientific names as well the bioactive components of selected spices.

Sl. No.	Common name	Scientific name	Bioactive components
1.	Turmeric	*Curcuma longa*	curcumin
2.	Cinnamon	*Cinnamomum zeylanicum*	cinnamaldehyde
3.	Black pepper	*Piper nigrum*	piperine, alpha-pinene
4.	Asafoetida	*Ferula asafetida*	alpha-pinene, diallyl-disulfide, ferulic acid, luteolin
5.	Bay leaves	*Laurus nobilis*	alpha-pinene
6.	Black cumin	*Nigella sativa*	thymoquinone
7.	Sage	*Salvia officinalis*	carnosol
8.	Fennel	*Foeniculum vulgare*	quercetin
9.	Saffron	*Crocus sativus*	crocin
10.	Clove	*Syzygium aromaticum*	eugenol
11.	Caraway	*Carum carvi*	carvacrol
12.	Dried red chili	*Capsicum annuum L.*	capsaicin, ferulic acid
13.	Dried mango powder	*Mangifera Indica L.*	beta-carotene, gallic acid

**Figure 4:**
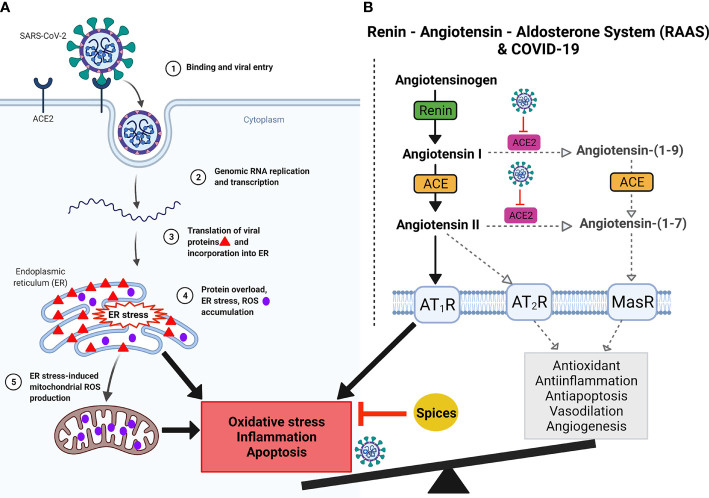
Graphical demonstration of predicted SARS-CoV-2 infection, ACE2 blockage, oxidative stress, and RAAS imbalance. **(A)** Binding of SARS-CoV-2 to ACE2 (1) followed by profuse replication, transcription and translation (2) of SARS-CoV-2 in the host cell leads to endoplasmic reticulum (ER) stress due to accumulation of newly synthesized viral proteins in the ER (3). Protein overload in the ER leads to reactive oxygen species (ROS) generation in the ER (4). Further, ER stress also induces mitochondrial ROS production (5). Together, ER stress and ROS accumulation lead to inflammation and apoptosis. **(B)** Renin, angiotensin and aldosterone together constitute the Renin-Angiotensin-Aldosterone System (RAAS) and its imbalances in SARS-CoV-2 infection. Angiotensinogen is produced in the liver and cleaved by renin to form angiotensin (I). Angiotensin I is converted to angiotensin II by angiotensin-converting enzyme (ACE). This conversion occurs in the lungs, where ACE is expressed by vascular endothelial and lung epithelial cells. During homeostasis, Angiotensin I is acted upon by ACE2 to generate Angiotensin-(1-9), which is cleaved by ACE to generate Angiotensin-(1-7). Further, ACE2 also cleaves Angiotensin II into Angiotensin-(1-7). Angiotensin-(1-7) *via* Mas receptor mediates antioxidative and anti-inflammatory responses. Angiotensin II binds to one of two G-protein coupled receptors, the AT_1_ and AT_2_ receptors. SARS-CoV2 binding to its potent ACE2 receptor diminishes ACE2 function, skewing the ACE/ACE2 equilibrium to a predominant pro-inflammatory ACE-Ang II-AT_1_R axis signaling. Imbalances in the RAAS system can alter the AT_2_R -MasR pathway, which is very important for vasodilation, angiogenesis, anti-inflammatory, antioxidative, and antiapoptotic activities. In contrast, AT_1_R pathway is important for vasoconstriction, inflammation, oxidative stress, and apoptosis. Virus binding to ACE2 dysregulates AT_2_R-MasR pathway. Imbalances of ACE and ACE2 generate ROS, leading to oxidative stress, which causes hyperinflammation. In addition, hyperinflammation with cellular oxidative stress imbalances causes disease severity and when reaches its climax leads to respiratory failure as discussed in **Figures 1** and **5**. Spices could potentially inhibit inflammation and restore homeostasis. Created with BioRender.com.

Virus infection and replication, including SARS-CoV-2, impairs redox potential inside the cells, mediating inflammation (Silvagno et al., 2020; Sun et al., 2020). Explosive replication, transcription, and translation of SARS-CoV-2 in the host cell leads to endoplasmic reticulum (ER) stress due to the accumulation of newly synthesized viral proteins in the ER. Protein overload in the ER leads to reactive oxygen species (ROS) generation in the ER (Chaudhari et al., 2014; Aoe, 2020). Further, ER stress also induces mitochondrial ROS production. Together, ER stress and ROS accumulation lead to inflammation (**Figure 4**). A recent study showed that SARS-CoV-2 could potentially utilize the ROS pathway to modulate the immune response (Wenzhong and Hualan, 2021). Furthermore, ACE2, the receptor for SARS-CoV-2, plays a key role in the renin-angiotensin-aldosterone system (RAAS) that regulates salt concentration and body fluid balance to maintain blood pressure. Evidence from related SARS-CoV studies suggests that by binding to ACE2, SARS-CoV-2 can also downregulate ACE2 impairing ACE/ACE2 balance and leading to oxidative stress, inflammation, and apoptosis (**Figure 4**) (Kuba et al., 2005; Ji et al., 2020; Silvagno et al., 2020; Xu et al., 2020). Thus, the balance between oxidative and antioxidative pathways plays a significant role in protecting against viral-induced tissue damage.

**Figure 5:**
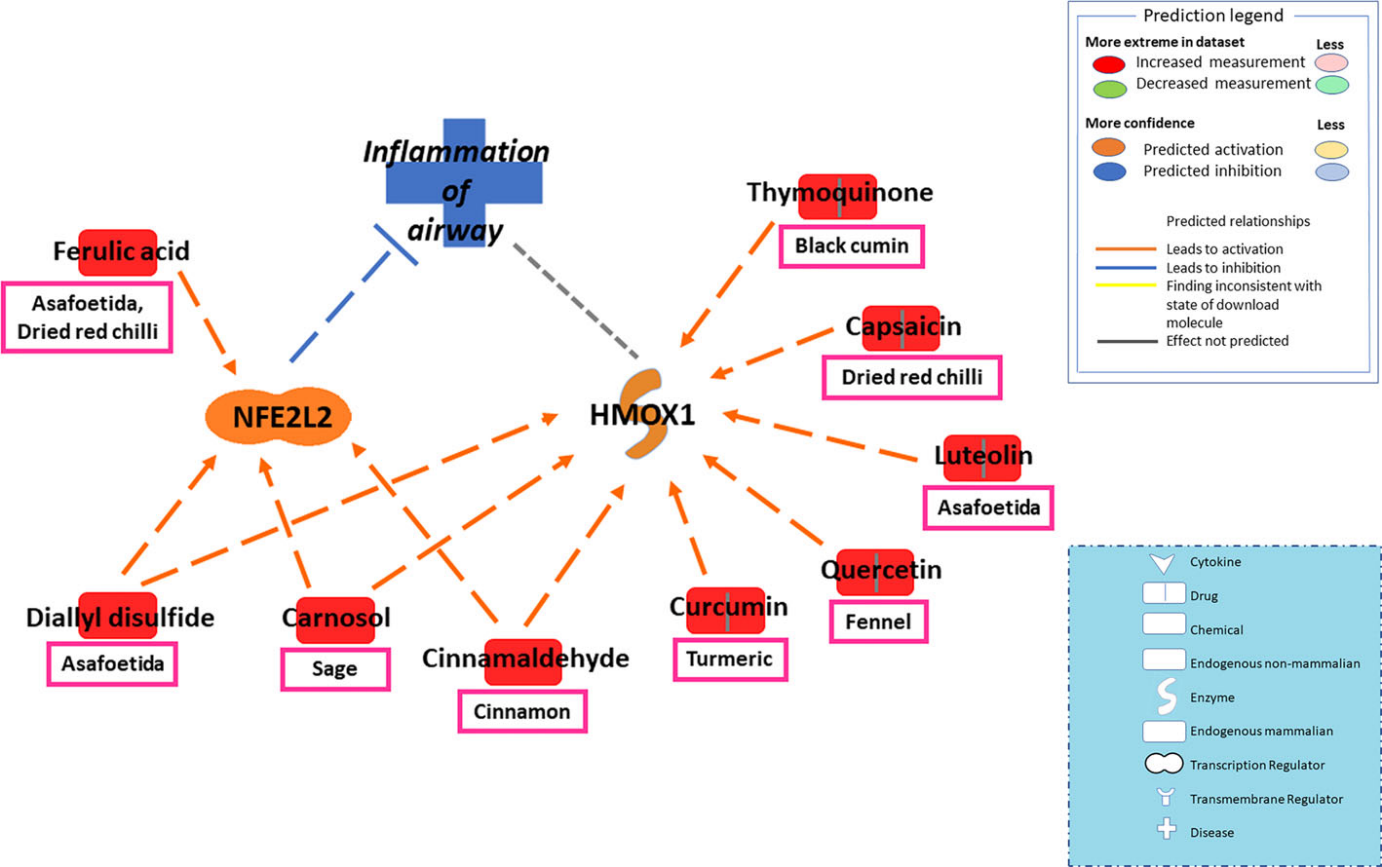
Network depicting interactions between bioactive components in spices and major antioxidant mediators NFE2L2 (Nrf2) and Hmox1 in alleviating oxidative stress-induced airway inflammation. The balance between oxidative and antioxidative pathways plays a significant role in protecting against viral-induced tissue damage. In the molecular mechanism of antioxidant pathways, Nrf2 (nuclear factor erythroid 2-related factor 2) is a major antioxidant molecule that regulates a wide array of genes involved in oxidative stress and inflammation. The antioxidant gene Heme oxygenase 1 or Hmox1 also lies downstream of Nrf2. Spice-derived bioactive components such as ferulic acid, cinnamaldehyde, carnosol, and diallyl disulfide could activate Nrf2, which in turn could attenuate oxidative stress-induced lung damage. Furthermore, Hmox1 can also be activated by several bioactive components, including Nrf2 activators- cinnamaldehyde, carnosol, and diallyl disulfide. The dashed line is a potential prediction for interaction between Hmox1 and airway inflammation.

To take it further, IPA screened several bioactive components of spices that can potentially inhibit oxidative stress and restore RAAS homeostasis. In the molecular mechanism of antioxidant pathways, Nrf2 (nuclear factor erythroid 2-related factor 2) is a major antioxidant molecule that regulates a wide array of genes involved in oxidative stress (such as antioxidant gene Heme oxygenase 1 or Hmox1) and inflammation (Tonelli et al., 2018). By interacting with Nrf2 and Hmox1, spice-derived bioactive components such as ferulic acid, curcumin, cinnamaldehyde, carnosol, and diallyl disulfide could potentially regulate the ROS levels and attenuate oxidative stress-induced lung damage (**Figures 2** and **5**).

The antioxidant property of spices through activation of Nrf2 has also been discussed recently (Bousquet et al., 2020; Bousquet et al., 2021). Many bioactive components of spices included in this study (**Table 1**), such as capsaicin, curcumin, piperine, and quercetin, are known agonists of TRP (transient receptor potential) cation channels. TRPs are oxidative stress sensors and induces inflammation. It has been suggested that by desensitizing TRP channels, spices can likely reduce COVID-19 severity (Bousquet et al., 2020). The current study supports the antioxidative and anti-inflammatory role of spices in COVID-19 while providing clues about their possible mode of action.

Additionally, multidrug antimicrobial resistance is a major factor responsible for sepsis and septic shock. Several spices and their bioactive components that are screened in the current study (**Table 1**), such as black pepper, clove, cumin, fennel, and cinnamon, have demonstrated potent inhibitory effects in *in vitro* and *in vivo* studies against various pathogenic bacteria and fungi such as *E. coli*, *E. faecalis*, *S. aureus*, *S. typhimurium*, *S. dysenteriae*, *B. subtilis*, *K. pneumonia*, *A. nijer*, *A. parasiticus* and *C. albicans.* Cinnamon has shown prominent anti-biofilm activity against methicillin-resistant *S. aureus* (MRSA) strain (Singh et al., 2004; Schmidt et al., 2007; Allahghadri et al., 2010; Bisht et al., 2014; Cui et al., 2016). This finding suggests that spices are promising therapeutic options in managing non-SARS-CoV-2 sepsis and SARS-CoV-2 induced viral sepsis.

## Conclusion

Despite the availability of repurposed drugs and the development of vaccines, the emergence of SARS-CoV-2 mutant strains poses a significant challenge in limiting the spread of the virus. The four novel vaccines, BNT162b2 vaccine, mRNA-1273 vaccine, Ad26.COV2.S vaccine and ChAdOx1 nCoV-19 targets the RBD (receptor binding domain) site in the SARS-CoV-2 spike protein. The four variants of concern- alpha, beta, gamma, and delta- have developed RBD mutations that enhance virulence, transmissibility, and reduction in neutralization by antibodies produced in response to a vaccine (Cascella et al., 2021).

Antiviral drugs and monoclonal antibody therapies are effective during the early phase of infection, where the SARS-CoV-2 replication reaches its peak. Immunomodulatory agents may help during the later stage of COVID-19, which is associated with hyperinflammation. However, data from several trial studies have generated mixed results about their potential (Joyner et al., 2020; Simonovich et al., 2021). Results from the WHO SOLIDARITY Trial did not find remdesivir to significantly affect the overall hospital stay or mortality in COVID-19 patients (Zhang and Mylonakis, 2021). Other antiviral medicines proposed initially for COVID-19 such as hydroxychloroquine, lopinavir or ritonavir, ivermectin, oseltamivir, and amantadine did not improve the clinical status in randomized control trials and are currently not accepted for universal therapy. Given the complicated viral replication kinetics and cytopathy combined with aberrant inflammation, designing a therapeutic is challenging. Dietary sources can help overcome this challenge.

Spices and their bioactive components present an alternative potential therapeutic approach for hyperinflammation and hypercytokinemia observed in COVID-19. Spices are a product of nature, generally recognized as safe and consumed worldwide. While our findings focus on the immune-modulatory and beneficial additive cellular factors of spices that favor the host, molecular docking studies suggest that spices exhibit antiviral properties by inhibiting SARS-CoV-2 spike protein and main protease (M^pro^) (Kumar et al., 2020; Sen et al., 2020; Natesh et al., 2021). A recent randomized controlled trial study demonstrated that administration of curcumin in nano micelles form significantly decreases IL6 and IL-1β in COVID-19 patients (Valizadeh et al., 2020). This potential dual inhibitory effects of spices both on the virus entry or replication and the hyperinflammatory stage make it a promising therapeutic option against all stages of COVID-19. Additionally, it would be interesting to identify the potential inhibitory property of spices on other viral proteins that are involved in replication, transcription, and assembly processes such as membrane (M) protein, envelop (E) protein, nucleocapsid (N) protein, helicase and RNA-dependent RNA polymerase (RdRp).

The results of the past and current studies provide a proof of concept for the hypothesis that bioactive components of spices may provide a solution for COVID-19 prevention. However, the benefits of spices need to be assessed thoroughly in well-designed large patient cohorts and double-blinded placebo groups. It would be relevant to study the effect of spice-derived bioactive components on cytokine release by immune cells during SARS-CoV-2 infection as massive infiltration of macrophages and neutrophils are observed in hospitalized COVID-19 cases (Li et al., 2020). Also, the administration of bioactive components at different stages of disease needs assessment in suitable *in-vivo* models. Suitable animal models for SARS-CoV-2 are still in nascent stages, and the zoonotic potential of the virus limits wide laboratory application. Well-characterized murine models of mouse hepatitis virus (MHV), which belongs to the same family as SARS-CoV-2 and require only biosafety containment level 2, could be an effective and yet simpler surrogate system testing therapeutic strategies against hepato-neuro COVID (Korner et al., 2020). It is worth noting that the timing of the administration of immune modulators is crucial. IL6 is one of the cytokines shown to be elevated in COVID-19 patients, and it is also a vital cytokine needed to mount a preliminary immune response against virus infection. Loss of IL6 results in increased persistence of influenza virus in the lungs and death in mice (Dienz et al., 2012). *In vitro* and *in vivo* studies can be designed to assess whether active ingredients from spices could help survive lymphocytes during SARS-CoV-2 infection and prevent lymphocytopenia associated with secondary infections. Future research directed at better understanding the mechanism of action or deciding the stage during which spice-based bioactive components could prove efficient in maintaining cytokine balance needs to be encouraged. Research involving both *in vitro* and *in vivo* experimental systems is required to determine the actual translational values of spices as immunity boosters against SARS-CoV-2 and emerging coronavirus threats. In addition, future studies can also investigate the feasibility of combining spice-derived bioactive components with standard medicines as multiple target therapy will be more efficient in battling SARS-CoV-2 infection than single-drug treatment.

## Data Availability Statement

The original contributions presented in the study are included in the article/supplementary material. Further inquiries can be directed to the corresponding author.

## Author Contributions

SS drafted the paper and helped with the literature search. DB, GK, SK, and OS did the literature search, helped prepare figures, edited and revised the manuscript. AG assisted in the literature search. SA and SS prepared the figure. SS and JD put forward the hypothesis. JD conceptualized, supervised the execution of the work, and critically revised the manuscript. All authors contributed to the article and approved the submitted version.

## Funding

SS received a fellowship from IISER-K, DB was given fellowship from Ministry of Education, India; University Grants Commission (UGC), and Council of Scientific and Industrial Research (CSIR) provided fellowships to GK and SK.

## Conflict of Interest

The authors declare that the research was conducted in the absence of any commercial or financial relationships that could be construed as a potential conflict of interest.

## Publisher’s Note

All claims expressed in this article are solely those of the authors and do not necessarily represent those of their affiliated organizations, or those of the publisher, the editors and the reviewers. Any product that may be evaluated in this article, or claim that may be made by its manufacturer, is not guaranteed or endorsed by the publisher.
